# A Decision-Support Software to Improve the Standard Care in Chinese Type 2 Diabetes

**DOI:** 10.1155/2019/5491743

**Published:** 2019-11-11

**Authors:** Yingying Luo, Yongzan Zhu, Jing Chen, Xueying Gao, Wenjia Yang, Xiantong Zou, Xianghai Zhou, Linong Ji

**Affiliations:** ^1^Department of Endocrinology and Metabolism, Peking University People's Hospital, China; ^2^Peking University Diabetes Center, Peking University Health Science Center, China; ^3^Academic Affair Department, Chinese Medical Association, China

## Abstract

**Background:**

To develop a decision-support software according to the Chinese Diabetes Society guideline in order to improve the standard care in type 2 diabetes.

**Methods:**

Firstly, we developed a decision-support software for healthcare professionals. It was an independent software on a tablet to record the data of patients and treatments given by their physicians. A major function of the software was to remind doctors when and how they should implement the standard care as recommended by the Chinese Diabetes Society guideline. Secondly, we compared the baseline data of standard care including statin and aspirin usage with data from a previous “3B study” to see whether there was an improvement of these standard cares. Finally, we further compared the data during four quarters of the whole year to evaluate whether there was a continuous improvement.

**Results:**

During the first quarter, 27,291 cases and 27,352 cases were collected with complete information about statin and aspirin usage, respectively. The percentage of patients treated with statins and aspirin in our study was significantly higher than that reported in the 3B study (59.6% vs. 19.9% and 59.8% vs. 18.5%, *P* < 0.001). There were no significant differences among the four quarters for the percentage of the patients who were taking statin or aspirin (*P* > 0.05).

**Conclusion:**

Our decision-support software has been shown to be effective in continuously improving the standardization of comprehensive treatment in type 2 diabetes.

## 1. Background

Modern medicine has achieved a remarkable success and has contributed greatly to health and longevity, but there is a big gap between the ideal world where most of the medical science research is conducted and the real world in medical care where the knowledge gained from medical science research is implemented and tested. In addition, medical harm caused by healthcare is one of the major avoidable harms [1, 2]. There is a huge and unexpected variability in the quality of medical care delivered to patients in daily medical practice [3].

Back in 2003, a report from the Institute of Medicine has raised the concern that priority should be given to the “improvement of healthcare quality” at the national level [4]. There were 20 clinical topics listed in this report. For every topic, there were “best medical cares” supported by sufficient clinical evidence. Unfortunately, the implementation of all the “best medical cares” was really disappointing, at a cost of thousands of lives every year at that time [5].

China is the country with the largest diabetic population [6]. The explosion in the number of people affected by diabetes has created a huge and debilitating burden of death and disability. Numerous clinical studies all over the world have produced lots of evidence for “best medical care” in diabetes management, and almost all of these “best medical cares” were recommended by diabetes guidelines worldwide. For example, the use of aspirin and statins could decrease the risk of both cardiovascular disease and mortality [7–9]. Regular eye examination annually could decrease the risk of developing blindness [10–12]. Feet examination could decrease the risk of lower-extremity amputation [13]. We call all of these strategies “standard care” in type 2 diabetes. Guidelines from many academic organizations have recommend implementing all these standard cares during our daily clinical practice [14, 15].

However, most of the “best medical cares” were not practiced in daily diabetes care in China. For example, the “3B study” was a national observational study to assess the level of control of blood glucose, blood pressure, and blood lipids (3Bs) among patients with type 2 diabetes [16]. The results from this study had shown that only 5.6% of the 25,817 type 2 diabetes patients surveyed in this study had met all recommended targets (defined as HbA1c < 7%, total cholesterol < 4.5 mmol/L, and blood pressure < 130/80 mmHg, respectively) for blood glucose, blood lipid, and blood pressure control. In this study, the proportion of patients who were taking metformin which was the first-line antidiabetic drug was less than 40%. Only 19.9% of the patients were treated statins, and even less (18.5%) were on aspirin therapy.

Therefore, it is of great urgency to improve the quality of diabetes care and allow all diabetic patients to benefit from the fruit of medical science in China.

The aim of our study was to develop a decision-support software according to the Chinese Diabetes Society guideline and, with this software as the core of the instruments, develop a set of tools for both physicians and patients to improve the quality of healthcare in type 2 diabetes [17]. To evaluate the efficacy of this software, we would further record the daily clinical practice of physicians from 72 clinical sites during a one-year period of time using this software and finally build a dataset to see whether there is an improvement of the implementation of standard care of type 2 diabetes.

In our previous study, we have shown that we have improved the standardization of metformin use in Chinese type 2 diabetes patients with this software [17]. Therefore, we would like to further evaluate whether statin and aspirin usage could be improved with this software as well.

## 2. Methods

### 2.1. Study Design

The following three parts were included in our study: (1) Development of the decision-support software: for healthcare professionals, a decision-support software was built. It was an independent software based on a tablet to record the data of patients and the treatments given by their physicians. Besides recording the data, the other major function of the software was to remind the doctors when and how they should implement the standard care (metformin as a first-line antihyperglycemic drug, blood pressure control, statin use, and aspirin treatment) to their patients as recommended by the Chinese Diabetes Society in their guideline published in 2010. (2) Comparison between the baseline data of standard care including statin and aspirin usage as well as metformin treatment which was published previously with data from a study named “3B study”: we compared the baseline data we collected with our software during the first quarter of the study with data from the previous “3B study” to see whether there was an improvement in the implementation of the standard care in type 2 diabetes. (3) Data analysis and comparison among four quarters: we further compared the data during the four quarters of the first year to evaluate whether there was a continuous improvement in the implementation of all these standardized cares.

### 2.2. Development of Quality Improvement Tools

We developed a set of tools to improve the quality of healthcare in patients with type 2 diabetes.

For healthcare professionals, a decision-support software was built. The key concept of the software is to support the physicians to make the decision to implement the standard care to type 2 diabetes patients according to the Chinese Diabetes Society guideline. Besides the software, we also designed a table board. Doctors might put this table board on their table, and all the standard cares recommended in the guideline can be clearly seen. On this table board, there were two tables and three flowcharts. In the first table, the targets of all the metabolic parameters for patients with type 2 diabetes were listed. In the second table, a detailed visit plan including a timetable and contents of each visit was listed. The first flowchart was the algorithm for an antihyperglycemic pathway. The second flowchart included an antihypertensive algorithm and recommendations for the use of statins and aspirin. The third flowchart was about when and how to screen for neuropathy in diabetic patients.

For patients with type 2 diabetes, we designed a “passport” for them. There were three major parts in the passport. The first part was about their targets of disease control including the ideal body weight, smoking cessation, targets of HbA1c, blood pressure, lipid, and the frequency of receiving advice when screening for complications. The second part was the data record. Patients may record their physical and laboratory examination results in this part. They could also record their treatment and compare the difference between their own therapy and the standard care recommended in the guideline. The algorithm for an antihyperglycemic treatment in the guideline was also printed in this part. The third part included some key knowledge about diabetes, including what diabetes was, the risk factors of developing diabetes, and how to control the disease.

### 2.3. Data Collection

At least 200 endocrinologists from 72 hospitals all over the country would be recruited. They were asked to use the quality improvement instruments mentioned above and collect data with the software we have developed. In the outpatient clinic, data of the first five patients they met would be recorded. They were also asked to record information from at least 30 patients in the ward every month. The study would last for one year. After one year, the rate of the implementation of each standard care would be calculated and compared with the baseline data which was taken from a previous study and with the data we collected during the first three months of this study.

### 2.4. Statistical Methods

SPSS18.0 was used for data analysis. Variables with normal distribution were indicated as mean ± standard deviation. Variables with abnormal distribution would be logarithmically transformed before analysis. An independent sample *t*-test was used to compare means between two groups. A Chi-square test was performed to compare rates among groups.

## 3. Results

### 3.1. Development of the Software

We developed the software according to the Chinese Diabetes Society guideline for type 2 diabetes published in 2010. Because different electronic record systems were used in different hospitals in China, at the first step, this software was not combined with the electronic record system. It was an independent software based on a tablet to record the data of patients and treatments given by their physicians. When doctors went to the page to record the antihyperglycemic treatments they gave to the patients, they would see the algorithm provided by the Chinese Diabetes Society (see Supplementary [Supplementary-material supplementary-material-1]). The key concept in this page was to remind doctors that metformin should be the first-line therapy if the patient did not have any contraindications. If the doctors did not choose metformin as the first drug they used, a dialog box would appear on the screen to ask the doctors to record the reasons why they did not choose metformin for the patients. After they finish recording the antihyperglycemic treatments, doctors would go to the next two pages to record the use of statins and aspirin of their patients. On these two pages, the contraindications and indications of these two drugs would appear to help doctors to make the decision whether they would prescribe these two drugs to their patients. Furthermore, the risk factors of cardiovascular disease of each patient would be extracted automatically from the patient's data which had been recorded previously. Finally, antihypertensive treatment would be recorded. The blood pressure of each patient would also be extracted from previous data. If the patient's blood pressure was over 140/80 mmHg, the software would recommend to the doctors to either give some advice on lifestyle modification or further prescribe some antihypertensive medications. There would also be a dialog box that would appear on the screen to give advice on the use of ACE inhibitors or ARBs as the preferred drugs.

### 3.2. Comparison between the Baseline Data from Our Study and the Data from the Previous “3B Study”

#### 3.2.1. Statin Usage as the Secondary Prevention Treatment for Cardiovascular Disease

During the first quarter, 27,291 cases were collected with complete information about statin usage with our software. Among all these patients, 14,231 were already on statin treatment and 2038 were newly treated with statins. In total, 16,269 (59.6%) patients were on statin treatment. In the previous 3B study, only 19.9% of all the patients were taking statins. The percentage of patients treated with statins in our study was significantly higher than that in the 3B study (*P* < 0.001). Among those who have already had a cardiovascular event, 5188 of 5643 patients were treated with statins, which was significantly improved compared with the 3B study (91.8% vs. 34.9%) (see Table 1).

#### 3.2.2. Aspirin Usage as the Secondary Prevention Treatment for Cardiovascular Disease

During the first quarter, 27,352 cases were collected with complete information about aspirin usage with our software. Among all these patients, 13,376 were already on aspirin treatment and 2985 were newly treated with aspirin. In total, 16,361 (59.8%) patients were taking aspirin. In the previous 3B study, only 18.5% of all the patients were taking aspirin. The percentage of patients treated with aspirin in our study was significantly higher than that in the 3B study (*P* < 0.001). Among those who have already had a cardiovascular event, 5427 of 6475 patients were treated with aspirin, which was significantly improved compared with the 3B study (83.8% vs. 39%) (see Table 2).

### 3.3. Continuous Improvement of Standard Care Was Seen during the Whole Year

#### 3.3.1. Statin

**Figure 1:**
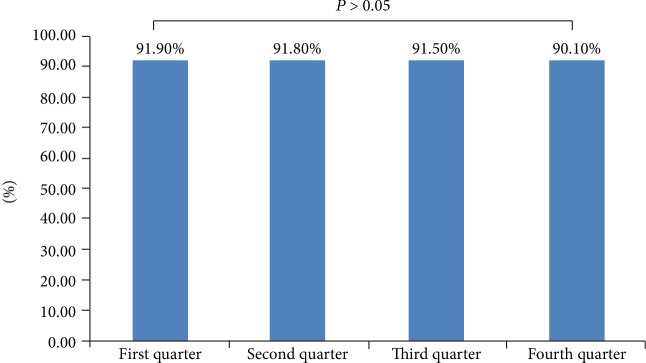
Comparison of statin treatment as a secondary prevention strategy during the whole year in our study.

During the first year of our study, 30,449 cases were collected with complete information about statin usage with our software. The percentage of patients who were taking statins as a secondary prevention strategy during the four quarters were 91.9%, 91.8%, 91.5%, and 90.1%. There was no significant difference among the four quarters (*P* > 0.05) (see Figure 1).

#### 3.3.2. Aspirin

**Figure 2:**
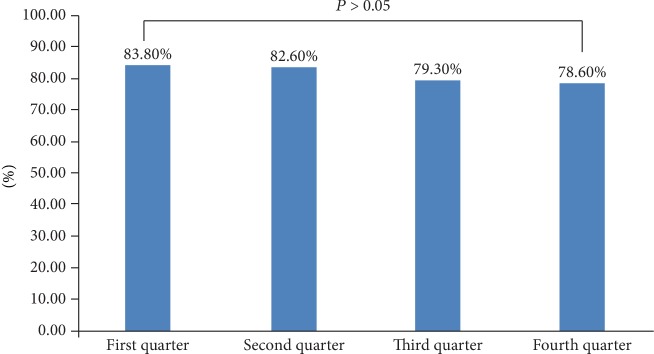
Comparison of aspirin treatment as a secondary prevention strategy during the whole year in our study.

During the first year of our study, 34,939 cases were collected with complete information about aspirin treatment with our software. The percentage of patients who were taking aspirin as a secondary prevention strategy during the four quarters were 83.8%, 82.6%, 79.3%, and 78.6%. There was no significant difference among the four quarters (*P* > 0.05) (see Figure 2).

## 4. Discussion

A previous study [16] has revealed big gaps between real world practice and clinical guidelines. How to close the gap and improve the quality of diabetes care in China is a key issue of great importance. From numbers of clinical trials, evidence for “standard medical care” in diabetes management has been built and has been proven to be effective. Epidemiological studies also showed that the implementation of these standard care of diabetes could improve the quality of medical care and could reduce the morbidity and mortality of diabetic patients [18, 19].

In 2012, a study entitled “Trends in Death Rates among U.S. Adults with and without Diabetes between 1997 and 2006: Findings from the National Health Interview Survey” was published in “Diabetes Care” [18]. In this study, the authors used data from the National Health Interview Surveys linked to the National Death Index and have compared 3-year death rates of four consecutive nationally representative samples (1997–1998, 1999–2000, 2001–2002, and 2003–2004). They found that between the earliest and latest samples, among diabetic adults aged over 18 years old, the death rate of cardiovascular disease declined dramatically up to 40% (95% CI 23–54) and all-cause mortality declined by 23% (10–35) as well.

Similar with this study, Gregg et al. published another paper entitled “Changes in Diabetes-Related Complications in the United States, 1990–2010” on “New England Journal of Medicine” in 2014 [19]. In their study, they also showed encouraging results. Data from the National Health Interview Survey, the National Hospital Discharge Survey, the U.S. Renal Data System, and the U.S. National Vital Statistics System were used in this study. The authors compared the incidences of lower-extremity amputation, end-stage renal disease, acute myocardial infarction, stroke, and death from hyperglycemic crisis between 1990 and 2010, with age standardized to the U.S. population in the year 2000. Rates of all five complications declined between 1990 and 2010. Acute myocardial infarction and death from hyperglycemic crisis were the top two complications which declined the most (-67.8% and -64.4%, respectively). Stroke and amputations declined by approximately half. End-stage renal disease was the one with the smallest decline (-28.3%).

These findings all reflect a quality improvement of medical care in the U.S. In the late 1990s, DCCT and UKPDS study groups published their results. Since then, interventions to multiple risk factors were given much more emphasis which led to a better risk factor control, including blood pressure control, lipid control, and glucose control. This is why we saw the reduction of all complications of diabetes.

Improvement science is an emerging field, in which it shares common aspects with other areas of research such as implementation science, translational science, and knowledge translation. All of these fields are similar in their focus on translating what is learned from research into actual practice to improve care and outcomes. Improvement science is the study of factors that can encourage or impede improvement in the workplace. It is relatively new in medicine, but it has been used for some decades in other sectors, such as manufacturing, and particularly in the U.S. The goal of improvement science is making sure health services operate in a way that makes it possible to offer the best clinical care and thus improve people's health. Besides, improvement science also enables evidence-based practice to become routine practice. Our study is such a study that uses the concept of “improvement science” to improve the quality of diabetes care in China.

## 5. Conclusion

Our study is the first quality improvement study in China to fill the gap between guideline recommendations and real world practice in diabetes. From our study, we have developed a set of quality improvement instruments. We have also established an effective and applicable pathway to improve the quality of healthcare. Considering the large number of patients with type 2 diabetes in China, our study will bring an important and huge impact on disease control and minimize the burden of diabetes to both the family and the society. In the future, we could further modify this software and combine it into the hospitals' electronic record system to make it more practical and easier to use for daily clinical practice.

## Figures and Tables

**Table 1:** Comparison of the baseline data of statin usage from our study with that of the previous 3B study.

	Baseline data in our study	Data in the 3B study	*P* value
Statin usage in the whole population	59.6%	19.9%	*P* < 0.001
Statin usage in patients with CVD or stroke	91.9%	34.9%	*P* < 0.001

**Table 2:** Comparison of the baseline data of aspirin usage from our study with that of the previous 3B study.

	Baseline data in our study	Data in the 3B study	*P* value
Aspirin usage in the whole population	59.8%	18.5%	*P* < 0.001
Aspirin usage in patients with CVD or stroke	83.8%	39.0%	*P* < 0.001

## Data Availability

The data used to support the findings of this study are available from the corresponding author upon request.

## Abbreviations

BMI: Body mass index
HbA1c: Hemoglobin A1c
ACE inhibitor: Angiotensin-converting-enzyme inhibitor
ARB: Angiotensin II receptor blocker
DCCT: Diabetes Control and Complications Trial
UKPDS: United Kingdom Prospective Diabetes Study

## Supplementary Materials

Supplementary Materials Supplementary figure 1. The screen of the software: If the physician did not choose metformin as the first anti-diabetic drug: The software would ask the physicians whether they would prefer to use metformin as first line therapy automatically. If “yes”: metformin would be recorded as first line drug and be highlighted. If “no”: the physician must choose one of the reasons that they refuse to use metformin

## References

[1] de Vries E. N., Ramrattan M. A., Smorenburg S. M., Gouma D. J., Boermeester M. A. (2008). The incidence and nature of in-hospital adverse events: a systematic review. *Quality & Safety in Health Care*.

[2] Landrigan C. P., Parry G. J., Bones C. B., Hackbarth A. D., Goldmann D. A., Sharek P. J. (2010). Temporal trends in rates of patient harm resulting from medical care. *The New England Journal of Medicine*.

[3] Right Care (2011). *The NHS Atlas of Variation in Healthcare*.

[4] Adams K., Corrigan J. M., Institute of Medicine (US) Committee on Identifying Priority Areas for Quality Improvement (2003). *Priority Areas for National Action: Transforming Health Care Quality*.

[5] Shojania K. G., KM M. D., Wachter R. M., Owens D. K., AHRQ Technical Reviews (2004). *Closing the Quality Gap: A Critical Analysis of Quality Improvement Strategies (Vol 1: Series Overview and Methodology)*.

[6] Yang W., Lu J., Weng J. (2010). Prevalence of diabetes among men and women in China. *New England Journal of Medicine*.

[7] Antithrombotic Trialists' (ATT) Collaboration, Baigent C., Blackwell L. (2009). Aspirin in the primary and secondary prevention of vascular disease: collaborative meta-analysis of individual participant data from randomised trials. *The Lancet*.

[8] Pyorala K., Pedersen T. R., Kjekshus J., Faergeman O., Olsson A. G., Thorgeirsson G. (1997). Cholesterol lowering with simvastatin improves prognosis of diabetic patients with coronary heart disease. A subgroup analysis of the Scandinavian Simvastatin Survival Study (4S). *Diabetes Care*.

[9] Gaede P., Lund-Andersen H., Parving H. H., Pedersen O. (2008). Effect of a multifactorial intervention on mortality in type 2 diabetes. *New England Journal of Medicine*.

[10] Hooper P., Boucher M. C., Cruess A. (2012). Canadian Ophthalmological Society evidence-based clinical practice guidelines for the management of diabetic retinopathy. *Canadian Journal of Ophthalmology*.

[11] Agardh E., Tababat-Khani P. (2011). Adopting 3-year screening intervals for sight-threatening retinal vascular lesions in type 2 diabetic subjects without retinopathy. *Diabetes Care*.

[12] Ahmed J., Ward T. P., Bursell S. E., Aiello L. M., Cavallerano J. D., Vigersky R. A. (2006). The sensitivity and specificity of nonmydriatic digital stereoscopic retinal imaging in detecting diabetic retinopathy. *Diabetes Care*.

[13] Boulton A. J., Armstrong D. G., Albert S. F. (2008). Comprehensive foot examination and risk assessment: a report of the Task Force of the Foot Care Interest Group of the American Diabetes Association, with endorsement by the American Association of Clinical Endocrinologists. *Diabetes Care*.

[14] Chinese Diabetes Society (2014). Chinese guideline for prevention and treatment of type 2 diabetes (2013 Edition). *Chinese Journal of Diaetes Mellitus*.

[15] Association AD (2014). Standards of medical care in diabetes—2014. *Diabetes Care*.

[16] Ji L., Hu D., Pan C. (2013). Primacy of the 3B approach to control risk factors for cardiovascular disease among type 2 diabetes. *The American Journal of Medicine*.

[17] Luo Y., Chen J., Zhou X., Ji L. (2014). Decision-support software to improve the standardization of metformin treatment in type 2 diabetes. *Diabetes/Metabolism: Research & Reviews*.

[18] Gregg E. W., Cheng Y. J., Saydah S. (2012). Trends in death rates among U.S. adults with and without diabetes between 1997 and 2006: findings from the National Health Interview Survey. *Diabetes Care*.

[19] Gregg E. W., Li Y., Wang J. (2014). Changes in diabetes-related complications in the United States, 1990–2010. *New England Journal of Medicine*.

