# Supplementary material for: A Decision-Support Software to Improve the Standard Care in Chinese Type 2 Diabetes
Source: J Diabetes Res. 2019 Nov 11;2019:5491743. doi: 10.1155/2019/5491743 (PMC6881560; doi:10.1155/2019/5491743)

Supplementary figure 1. The screen of the software: If the physician did not choose metformin as the first anti-diabetic drug: The software would ask the physicians whether they would prefer to use metformin as first line therapy automatically. If "yes": metformin would be recorded as first line drug and be highlighted. If "no": the physician must choose one of the reasons that they refuse to use metformin.

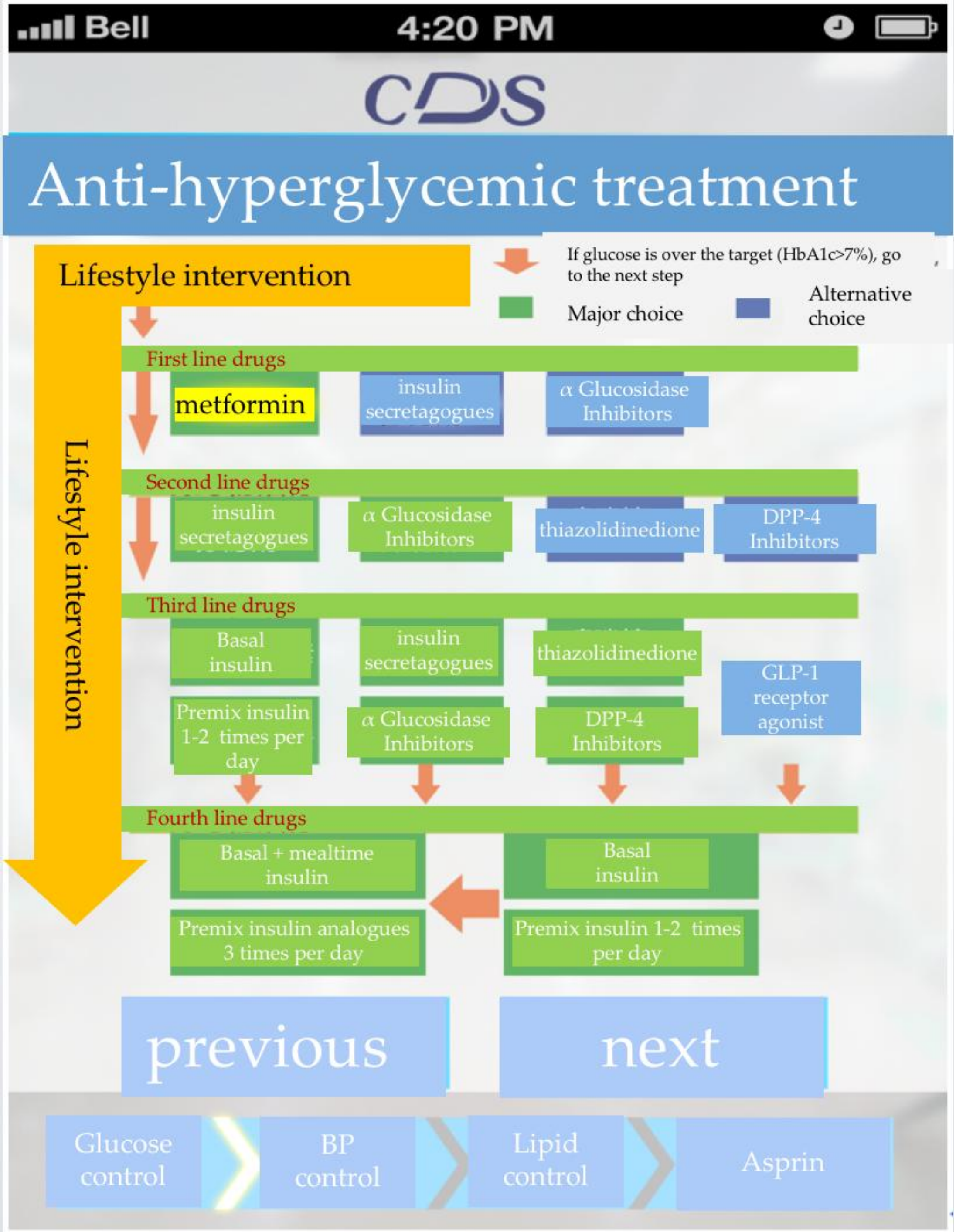

Supplement: Supplementary Materials — Supplementary figure 1. The screen of the software: If the physician did not choose metformin as the first anti-diabetic drug: The software would ask the physicians whether they would prefer to use metformin as first line therapy automatically. If “yes”: metformin would be recorded as first line drug and be highlighted. If “no”: the physician must choose one of the reasons that they refuse to use metformin [file 5491743.f1.pdf]
